# Prognostic significance of *CTNNB1* mutation in early stage endometrial carcinoma: a systematic review and meta-analysis

**DOI:** 10.1007/s00404-021-06385-0

**Published:** 2022-01-16

**Authors:** Antonio Travaglino, Antonio Raffone, Diego Raimondo, Sabrina Reppuccia, Alessandro Ruggiero, Alessandro Arena, Paolo Casadio, Fulvio Zullo, Luigi Insabato, Renato Seracchioli, Antonio Mollo

**Affiliations:** 1 Anatomic Pathology Unit, Department of Advanced Biomedical Sciences, School of Medicine, University of Naples Federico II, Naples, Italy; 2 Gynecology and Obstetrics Unit, Department of Neuroscience, Reproductive Sciences and Dentistry, School of Medicine, University of Naples Federico II, Via Sergio Pansini, 5, 80131 Naples, Italy; 3 Division of Gynaecology and Human Reproduction Physiopathology, Department of Medical and Surgical Sciences (DIMEC), IRCCS Azienda Ospedaliero-Univeristaria di Bologna. S. Orsola Hospital, University of Bologna, Via Massarenti 13, 40138 Bologna, Italy; 4 Gynecology and Obstetrics Unit, Department of Medicine, Surgery and Dentistry “Schola Medica Salernitana”, University of Salerno, 84081 Baronissi, Italy

**Keywords:** *β*-Catenin, CTNNB1, Endometrioid, Adjuvant treatment, Risk group

## Abstract

**Background:**

In the last years, mutations in the exon 3 of *CTNNB1* have emerged as a possible prognostic factor for recurrence in early stage endometrioid endometrial carcinoma, especially in cases with no specific molecular profile (NSMP).

**Objective:**

To define the prognostic value of *CTNNB1* mutations in early stage endometrioid endometrial carcinoma, through a systematic review and meta-analysis.

**Methods:**

Electronic databases were searched from their inception to November 2020 for all studies assessing the prognostic value of *CTNNB1* mutation in early stage (FIGO I–II) endometrioid endometrial carcinoma. Odds ratio (OR) for tumor recurrence and hazard ratio (HR) for disease-free survival (DFS) were calculated with a significant *p* value < 0.05.

**Results:**

Seven studies with 1031 patients were included. Four studies were suitable for meta-analysis of OR and showed significant association between *CTNNB1* mutation and the absolute number of recurrence (OR = 3.000; *p* = 0.019); the association became stronger after excluding patients with known molecular status other than NSMP (HR = 5.953; *p* = 0.012). Three studies were suitable for meta-analysis of HR and showed no significant association between *CTNNB1* mutation and decreased DFS (HR = 1.847; *p* = 0.303); the association became significant after excluding patients with known molecular status other than NSMP (HR = 2.831; *p* = 0.026).

**Conclusion:**

*CTNNB1* mutation is significantly associated with recurrence in early stage endometrioid endometrial carcinomas, especially in the NSMP, appearing potentially useful in directing adjuvant treatment.

## Introduction

Endometrial carcinoma (EC) is the most common gynecological malignancy in the western countries [1, 2]. The current European system for the prognostic stratification of EC is based on International Federation of Gynecology and Obstetrics (FIGO) stage and grade, histotype and lymphovascular space invasion (LVSI). Such system subdivides early stage EC (i.e., limited to the uterus) into 4 risk categories: low risk (FIGO IA G1–2 endometrioid with no substantial LVSI), intermediate risk (FIGO IB G1–2 endometrioid with no substantial LVSI; FIGO IA G3 endometrioid with no substantial LVSI; FIGO IA non-endometrioid without myometrial invasion), high–intermediate risk (Stage IA–B G1–2 endometrioid with LVSI; FIGO IB G3 endometrioid; FIGO II), high risk (FIGO III–IVA with no residual disease; FIGO I–IVA non-endometrioid with myometrial invasion and no residual disease) [3]. Such system can be integrated with the molecular classifier proposed by The Cancer Genome Atlas (TCGA), which identifies four prognostic groups: ultramutated/polymerase-*ε* (*POLE*) -mutant, hypermutated/microsatellite-instable, copy-number-low/no specific molecular profile (NSMP), and copy-number-high/*TP53*-mutant [4]. In fact, *POLE*-mutant ECs up to FIGO stage II are considered at low risk, while *TP53*-mutant endometrioid ECs are lumped together with non-endometrioid ECs; the other two TCGA groups are considered at intermediate prognosis and do not alter the risk category [3]. The main role of such stratification system is to guide adjuvant treatment. In fact, based on the risk category, patients may undergo observation alone, vaginal brachytherapy, external beam radiotherapy and/or chemotherapy [3].

However, the NSMP groups (which is the most represented TCGA group) appears to have a highly heterogeneous prognosis, which ranges from good (similar to that of the *POLE*-mutant group) to poor (similar to that of the *TP53*-mutant group) [5–10]. The overall good-to-intermediate prognosis is attributable to the high percentage of early stage endometrioid ECs with low-risk histological features within this group [11]. The NSMP group by definition lacks specific molecular signatures and is defined by the exclusion of the other TCGA group [4]. Therefore, the biological behavior of ECs within this group might be affected by other molecular features which are not considered in the TCGA classification. In recent years, several studies proposed that mutations in the exon 3 of *CTNNB1* (*β*-catenin-encoding gene) could identify low-grade, early stage endometrioid EC at increased risk of recurrence [12–16]. This would be of paramount importance for the patient management, in terms of choice of appropriate adjuvant treatment. However, other studies did not find the same results [17, 18], and most research groups do not take into account the *CTNNB1* status for the risk stratification of EC [19, 24].

The aim of this study was to assess the prognostic significance of *CTNNB1* mutation in early stage endometrioid EC, through a systematic review and meta-analysis.

## Materials and methods

### Study protocol

Based on previous studies [2, 25], we a priori designed methods for each review stage (electronic search, study selection, data extraction, risk of bias within studies assessment and data analysis), which was independently performed by two authors; disagreements were solved by consensus. This review was reported following the Preferred Reporting Items for Systematic Reviews and Meta-Analyses (PRISMA) guidelines [26].

### Search strategy and study selection

Four electronic databases (Scopus, Web of Sciences, Google Scholar and MEDLINE) were searched from their inception to November 2020 for all studies assessing the prognostic value of *CTNNB1* mutations in early stage (FIGO I–II) endometrioid EC. The following combination of text words was used: endometrial AND (cancer OR carcinoma) AND (CTNNB1 OR β-catenin). Reference lists of relevant studies were also searched. PICO [26] of our study were: “P” (population) = patients with early stage (FIGO I–II) endometrioid EC; “I” (intervention or risk factor) = *CTNNB1* exon 3 mutation; “C” (comparator) = *CTNNB1* wild type; “O” (outcome) = tumor recurrence or disease-free survival (DFS). The following exclusion criteria, defined a priori, were adopted: overlapping patient data, sample size < 10, data not extractable, reviews.

### Data extraction

Data from primary studies were extracted by two independent authors (SR and ARu) without modifications. Extracted data were: sample size, *CTNNB1* status and absolute number of recurrence (for the analysis of the absolute risk of recurrence), hazard ration (HR) for recurrence with 95% confidence interval (CI, for the analysis of DFS), selection criteria (i.e., FIGO grade and stage, histotype), period of enrollment, molecular methods and oncologic outcomes (for the risk of bias assessment); additional extracted data were patient age, body mass index (BMI) and follow-up duration.

### Risk of bias assessment

As previously described [25, 27], the risk of bias assessment was based on the revised Quality Assessment of Diagnostic Accuracy Studies (QUADAS-2) [28]. Four domains were assessed: (1) Patient selection (correct reporting of selection criteria and period of enrollment); (2) Index test (correct reporting of methods for *CTNNB1* mutation analysis); (3) Reference standard (correct reporting of oncologic outcomes); (4) Flow (inclusion of all eligible patients in the analysis). For each domain and in each study, the authors’ judgement was “low”, “unclear” or “high” risk of bias, as previously described [25, 27].

### Data analysis

For the studies that reported the absolute number of recurrence in *CTNNB*-mutant vs *CTNNB1*-wild-type cases, odds ratio (OR) was calculated to assess the association between *CTNNB1* status and recurrence. For the studies that performed Cox regression survival analysis for DFS, HR of *CTNNB1*-mutant cases compared to *CTNNB1*-mutant cases was assessed; only HRs from multivariate analyses (i.e., adjusted for other clinicopathological factors) were assessed. Statistical heterogeneity among studies was calculated using inconsistency index (*I*^2^) as previously described [2, 25]. A random-effect model was used to pool data. A *p* value < 0.05 was considered significant. Results of each study and pooled estimates were reported on forest plots with 95% CI.

Data analysis was performed using Comprehensive Meta-Analysis (Biostat,14 North Dean Street, Englewood, NJ 07631, USA).

## Results

### Study selection and characteristics

**Fig. 1:**
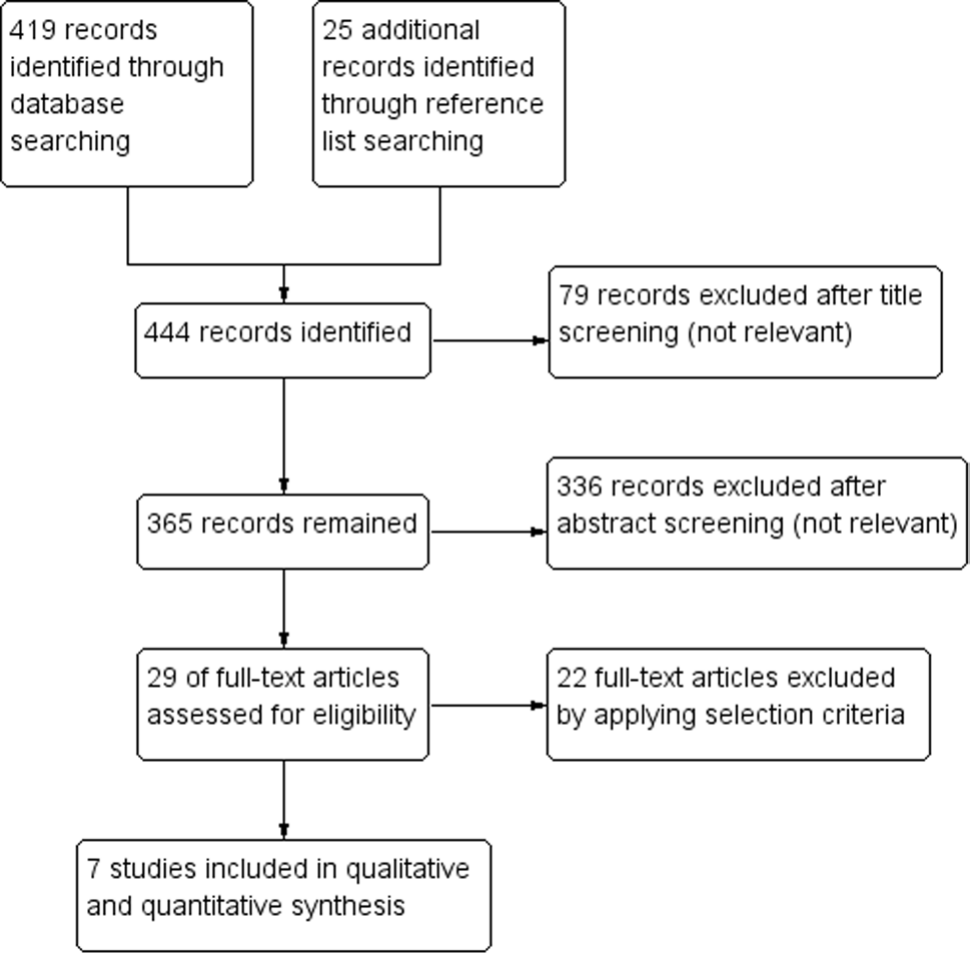
Flow diagram of studies identified in the systematic review (Prisma template [Preferred Reporting Item for Systematic Reviews and Meta-analyses])

Seven studies with a total sample size of 1031 patients with early-stage endometrioid EC were included [11–17]. The process of study selection is shown in Fig. 1. Regarding FIGO stage, two studies only included stage IA cases, four studies included stage IA–IB cases and one study included stage I–II cases. Regarding FIGO grade, three studies only included G1 cases, two studies included G1–2 cases and two studies also included selected subset of G3 cases; out of these G3 cases, endometrioid carcinomas with ≥ 50% myometrial invasion and non-endometrioid carcinomas were not considered. Mean patient age ranged from 57 to 69.6; mean BMI ranged from 28 to 34.2 and was not reported in three studies. Mean follow-up duration ranged from 33 to 131 months and was not reported in two studies. Adjuvant treatment included observation alone, vaginal brachytherapy and external beam radiotherapy. Characteristics of the included studies are shown in Table 1.

**Table 1:** Characteristics of the included studies

Study	Country	Period of enrollment	Selection criteria	Sample size	Age, mean/median, years	BMI, mean/median, kg/m^2^	Follow-up, mean/median, months	Adjuvant treatment (no.)	Recurrence (no.)
Myers 2014	USA	1998–2010	FIGO IA G1 EC	47	69.6 (cases) 62.8 (controls)	28 (cases) 34 (controls)	62 (cases) 124 (controls)	None (46), brachytherapy (1)	11
Stelloo 2016	Netherlands	1990–1997, 2000–2006	FIGO IB G1–2 EC, FIGO IA G3 EC	546 (443 were NSMP)	68	Not reported	131	None (241), brachytherapy (184), EBRT (409)	23 (only distant)
Kurnit 2017	USA	2000–2015	FIGO I–II G1–2 EC	125	60.6	33.8	Not reported	Not reported	Not reported
Imboden 2019	Switzerland	2002–2014	FIGO I G1–2 EC	41 (18 tested for CTNNB1)	67.8	Not reported	38.6 (cases) 93.5 (controls)	None (2), brachytherapy (16)	9
Moroney 2019	USA	2007–2017	FIGO I G1 EC	44	57 (cases) 59 (controls)	33.1 (cases) 34.2 (controls)	≥ 86 months	None	15
Li 2020	China	Not reported^a^	FIGO I^b^	294 (215 eligible for meta-analysis)	64	Not reported	Not reported	None (123), brachytherapy (92), EBRT (79)	Not reported
Stasenko 2020	USA	2009–2017	FIGO IA G1 EC	486 (36 tested for CTNNB1)	58	31	51 months (cases) 33 months (controls)	None	9

^a^Data are retrieved from the TCGA database

^b^The group of patients with high-risk features (i.e., G3 endometrioid with > 50% myometrial invasion and non-endometrioid) was excluded from meta-analysis

### Risk of bias assessment

**Fig. 2:**
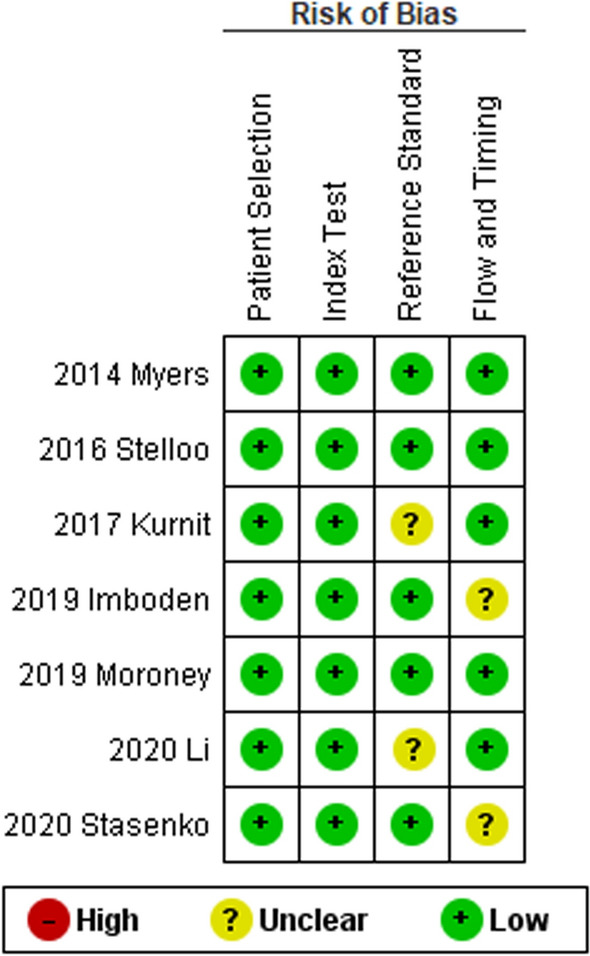
Assessment of risk of bias. Summary of risk of bias for each study; Plus sign: low risk of bias; minus sign: high risk of bias; question mark: unclear risk of bias

For the “Patient selection” domain, all studies were considered at low risk, since they reported period of enrollment and selection criteria; one study did not specify the period of enrollment but was still considered at low risk, since it assessed the TCGA database. For the “Index test” domain, all studies were considered at low risk, since they specified methods for *CTNNB1* assessment. For the “reference standard”, unclear risk of bias was assigned to two studies (follow-up data not clearly detailed). For the “flow” domain, two studies were considered at unclear risk, since they assessed *CTNNB1* mutations only in a subset of patient; the remaining studies were considered at low risk. Risk of bias assessment results are presented in Fig. 2.

### Meta-analysis

**Fig. 3:**
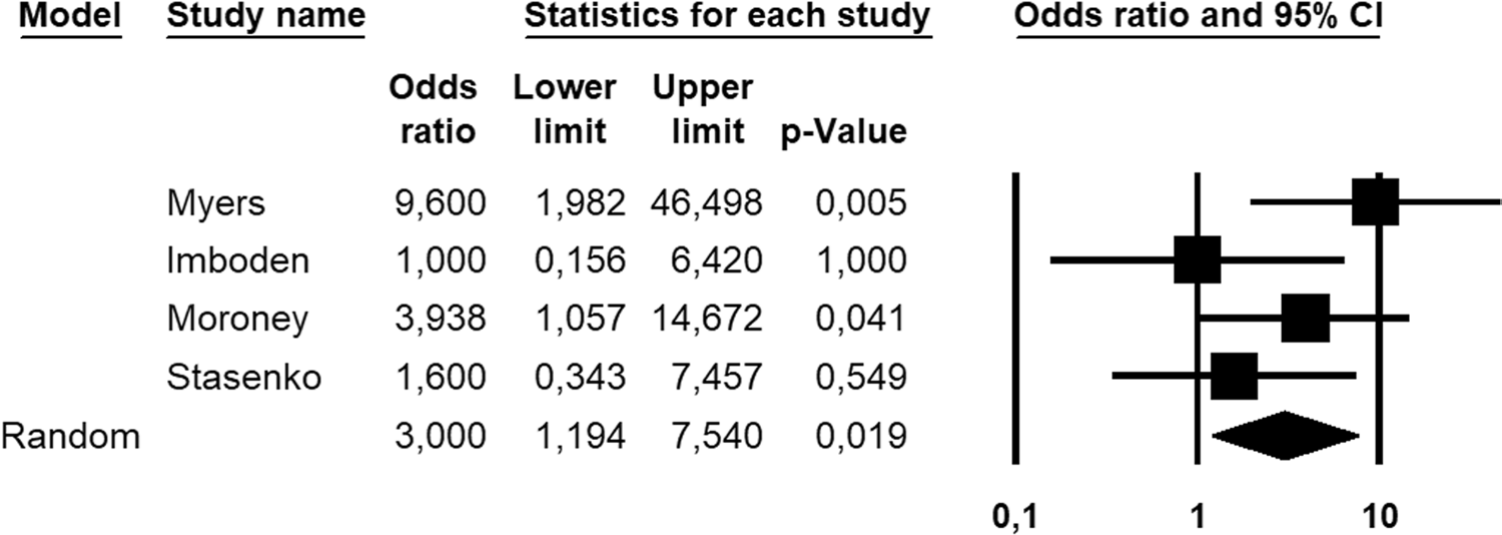
Forest plot reporting odds ratio (HR) values with 95% confidence interval (CI), for each study and as pooled estimate, for the association between *CTNNB1* mutation and endometrial cancer recurrence

**Fig. 4:**
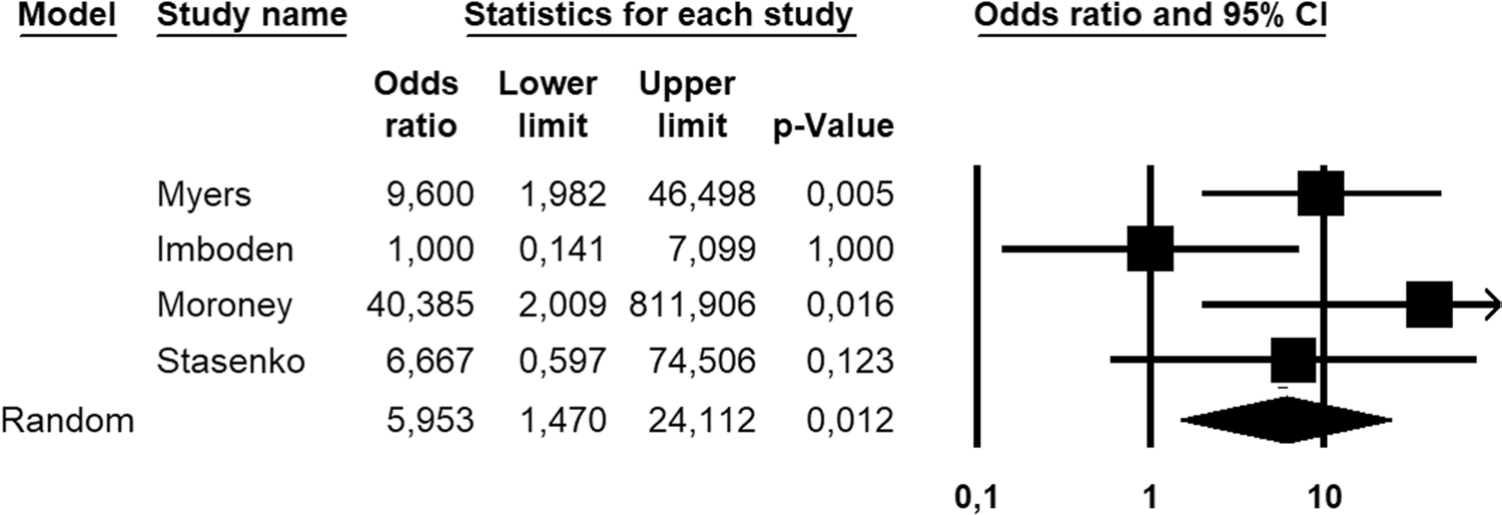
Forest plot reporting odds ratio (HR) values with 95% confidence interval (CI), for each study and as pooled estimate, for the association between *CTNNB1* mutation and endometrial cancer recurrence, after excluding the cases with known molecular status other than NSMP

One-hundred and forty-nine patients, all with FIGO stage I, low-grade (G1–2) EC, were assessed for the absolute number of recurrence; the DFS was not assessed in these patients. Overall, 44 patients (22.7%) recurred; *CTNNB1* mutations were detected in 23/44 (52.3%) recurrent patients and 26/101 (25.7%) non-recurrent patients. Statistical heterogeneity among studies was low (*I*^2^ = 29.1%, non-significant). Pooled OR was 3.000 (95% CI 1.194–7.540), indicating a significant association between *CTNNB1* mutation and recurrence (*p* = 0.019) (Fig. 3). The strength of association increased after excluding patients with known molecular status other than NSMP, with a pooled OR of 5.953 (95% CI 1.470–24.112; *p* = 0.012) (Fig. 4) and low statistical heterogeneity among studies (*I*^2^ = 41.2%).

**Fig. 5:**
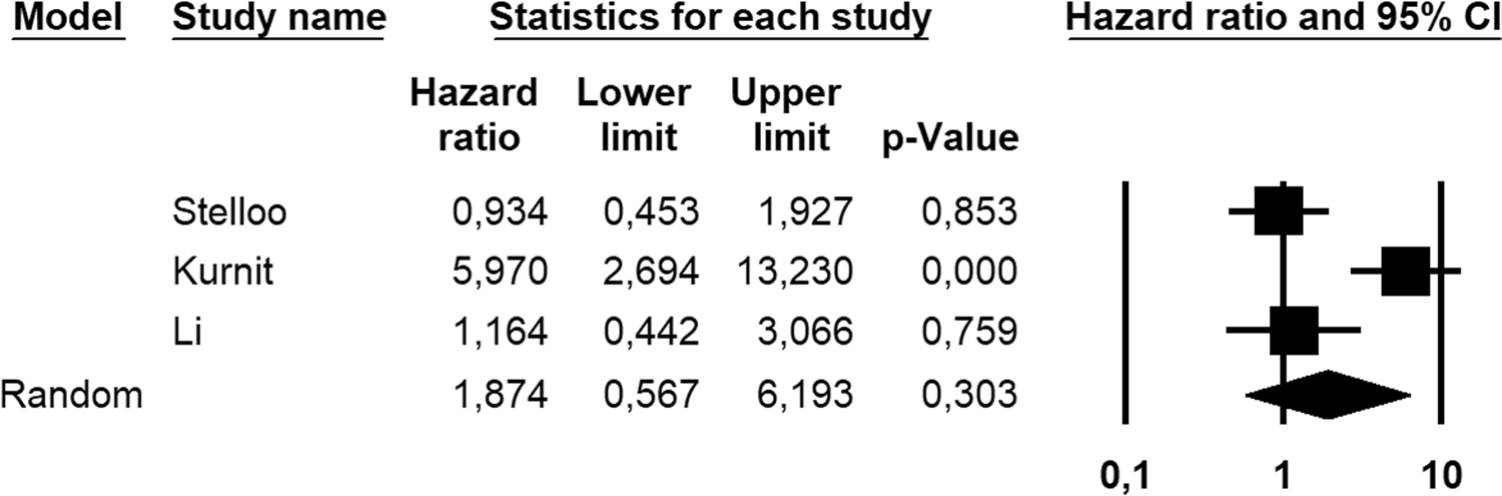
Forest plot reporting hazard ratio (HR) values with 95% confidence interval (CI), for each study and as pooled estimate, for the hazard of recurrence in *CTNNB1*-mutant vs *CTNNB1*-wild-type endometrial cancer

**Fig. 6:**
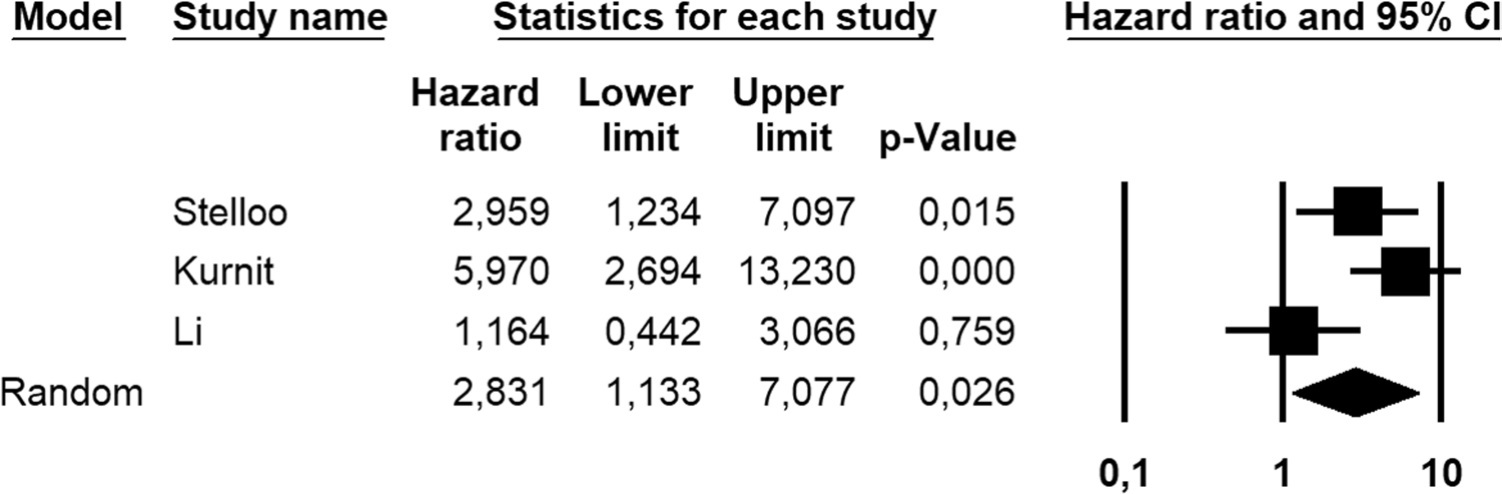
Forest plot reporting hazard ratio (HR) values with 95% confidence interval (CI), for each study and as pooled estimate, for the hazard of recurrence in *CTNNB1*-mutant vs *CTNNB1*-wild-type endometrial cancer, after excluding the cases with known molecular status other than NSMP

Eight-hundred and eighty-six patients were assessed for DFS; these patients also included FIGO stage II and G3 endometrioid EC with < 50% myometrial invasion. The absolute number of *CTNNB1*-mutant cases and recurrences was not available for these patients. Molecular status was known for 546/886 (61.6%) patients. Statistical heterogeneity among studies was high (*I*^2^ = 84.2%, significant). Pooled HR was 1.874 (95% CI 0.567–6.193), indicating no significant association between *CTNNB1* mutation and DFS (*p* = 0.303) (Fig. 5). After excluding patients with known molecular status other than NSMP, the association between *CTNNB1* mutation and decreased DFS became significant, with a pooled HR of 2.831 (95% CI 1.133–7.077; *p* = 0.026) (Fig. 6); statistical heterogeneity was moderate (*I*^2^ = 69.4%, significant).

## Discussion

This study showed that *CTNNB1* mutation was associated with increased absolute risk of recurrence in early stage endometrioid EC, especially after excluding patients with known molecular status other than NSMP; the association with DFS was significant only after excluding patients with known molecular status other than NSMP. To our knowledge, this is the first meta-analysis assessing the prognostic value of *CTNNB1* mutations in early stage endometrioid EC.

*CTNNB1* encodes for *β*-catenin, a key protein in the Wnt signaling pathway. In healthy tissues, *β*-catenin is normally expressed on the cell membrane, where it links cytoskeleton to cell–cell adherens junctions. As consequence of the Wnt pathway activation, *β*-catenin translocates into the nucleus, where it binds to transcription factors that favor cell proliferation. However, the accumulation of *β*-catenin into the nucleus may also occur in the case of pathologic alterations of the Wnt pathway, such *CTNNB1* mutation. These conditions are associated with carcinogenesis [29, 30]. In EC, the role of *CTNNB1* mutations is well documented, occurring in about 25–30% of cases. In particular, *CTNNB1* mutations are typically found in endometrioid histotype but not in serous histotype and are also observed in endometrioid intraepithelial neoplasia and atypical polypoid adenomyoma, which are regarded as precancerous lesions [4, 29–31].

In 2013, TCGA showed that *CTNNB1* mutations were particularly common in the NSMP group (about half of cases) [4]. The value TCGA molecular classification has repeatedly been confirmed in several subsequent studies [18–23]. However, it has also become evident that the NSMP group is too molecularly and prognostically heterogeneous to be clinically consistent. In fact, its prognosis strongly depends on FIGO grade and histotype [5, 10, 32]. The main issue is with early stage, low-grade endometrioid EC; in such subset of patients, identifying the cases at higher risk of recurrence which need adjuvant treatment appears crucial [3]. The Leiden group of the trans-PORTEC initiative proposed that *CTNNB1* mutations could identify these cases within the NSMP group. They found that, among EC with no specific molecular signature, *CTNNB1*-wild-type cases had a good prognosis similar to that of the POLE-mutant group, while *CTNNB1*-mutant cases had a prognosis intermediate between POLE-mutant cases and TP53-mutant cases, and similar to that of the microsatellite-instable group. They suggested that *CTNNB1*-mutant cases could be suitable to vaginal brachytherapy, while *CTNNB1*-wild-type cases could undergo observation alone [12]. The relevance of *CTNNB1* in early stage EC has also been advocated by other authors [11, 13–15]. In agreement with the findings of the Leiden group, Moroney et al*.* suggested that mismatch repair deficiency (a condition associated with microsatellite instability and a hypermutated phenotype [33, 34]) has a similar significance as CTNNB1 mutations in such subset of tumors [15].

To improve the clinical applicability of these findings, immunohistochemical surrogates of molecular markers have been tested and validated [33, 35]. Nuclear accumulation of *β*-catenin is the most obvious immunohistochemical surrogate of *CTNNB1* mutation. In endometrial lesions, there is a strong association between nuclear *β*-catenin and *CTNNB1* mutation. However, there are some limitations that impair the accuracy of *β*-catenin immunohistochemistry in this field. Indeed, sensitivity might be suboptimal, and the nuclear staining might be only focal and thus be missed [30, 36].

Although the clinical relevance of *CTNNB1* in early stage endometrioid EC is currently being assessed in prospective trials [37–39], most studies focused on the prognostic stratification of EC adopt the TCGA classification without considering *CTNNB1* mutations [19–24, 40, 41]. By pooling the published studies, we found that *CTNNB1* mutation was significantly associated with increased number of recurrences in early stage endometrioid EC; the association became stronger after excluding cases with known molecular status other than NSMP. On the other hand, the association between *CTNNB1* mutation and decreased DFS was not significant, although it became significant after excluding cases with known molecular status other than NSMP. Five out of seven included studies only included low-grade EC, which mostly fall into the NSMP subgroup, although individual studies also showed a significant prognostic value for *CTNNB1* mutation in subsets of high-grade EC [16]. These findings support that *CTNNB1* mutation may be a valuable marker for identifying early stage endometrioid EC which have increased risk of recurrence and need an adjuvant treatment. Our results, in agreement with the results of the trans-PORTEC group, suggest that *CTNNB1* may be prognostically valuable in NSMP cases. However, it should be remarked that the trans-PORTEC findings are mainly based on high–intermediate risk EC [13]. The significance of molecular features in the current ESGO low-risk category is less clear, and it has been suggested not to consider the TCGA classification in such category [42].

Interestingly, recent studies supported that the TCGA classification of endometrial carcinoma has a similar prognostic value in ovarian carcinoma; this finding was limited to endometrioid histotype, while high-grade serous carcinoma showed different prognostic marker and molecular signatures [43–47]. Since CTNNB1 mutations are also common in ovarian endometrioid carcinoma (even more than in its endometrial counterpart [43]), it cannot be excluded that CTNNB1 status might also be clinically useful in ovarian carcinoma.

Our results have some limitations which should be considered. First, not all studies performed the whole TCGA molecular classification, preventing to draw definitive conclusions about the significance of *CTNNB1* mutation in the different TCGA groups. Second, the primary studies selected cases at different FIGO stage (IA, IB, II), and it was, therefore, impossible to define whether the prognostic value of *CTNNB1* changed with different stages. Furthermore, other factors, such as the length of follow-up and the treatment adopted, may have affected the final results. We think that further studies in this field should assess the prognostic value of *CTNNB1* mutation in large cohorts stratified by both TCGA molecular groups and classical histopathological prognostic parameters (i.e., FIGO stage and grade, histotype, LVSI). Only in this way can the precise prognostic significance and clinical applicability of *CTNNB1* be defined.

## Conclusion

The presence of mutations in the exon 3 of *CTNNB1* appears as a significant predictor of recurrence in early stage endometroid EC, in particular in the NSMP EC. This finding strengthens the idea that *CTNNB1* status might be used to substratify early stage, low-grade EC with NSMP, aiding to identify cases that need adjuvant treatment. Further studies are encouraged to confirm these findings and define their clinical significance in cohorts of patients stratified by TCGA classification.
